# Analysis of myocardial temperature changes in conventional isolated coronary artery bypass grafting

**DOI:** 10.1007/s11748-014-0424-8

**Published:** 2014-12-01

**Authors:** Hiroshi Okamoto, Akinori Tamenishi, Toshihiko Nishi, Takao Niimi

**Affiliations:** Department of Thoracic and Cardiovascular Surgery, Yokkaichi Municipal Hospital, Shibata, Yokkaichi, 2-2-37 Japan

**Keywords:** Myocardial temperature, Cold blood cardioplegia, Coronary artery bypass grafting

## Abstract

**Backgrounds:**

To determine whether cold blood cardioplegia (CBCP) can get over coronary artery lesions, we analyzed the relationship between myocardial temperature changes and lesion severity of major coronary arteries.

**Methods and results:**

From April 1991 to October 2003, we measured myocardial temperature before and after antegrade and retrograde delivery of CBCP in 492 patients undergoing conventional coronary artery bypass grafting. Stenotic severity of three major coronary arteries was classified into four grades according to preoperative coronary arteriography; grade 0 for 50 % or less, 1 for 75 %, 2 for 90 %, 3 for 99 % or 100 %. We analyzed relationships between myocardial temperature changes [Δ*T*-A (antegrade) & Δ*T*-R (retrograde)] and the coronary artery lesion’s severity. Average Δ*T*-A of the right coronary artery had no relationship with stenotic grades. Mean Δ*T*-A of the left anterior descending (LAD) became less and less in proportion to its stenotic grade [9.7 °C for grade 0, 8.2 °C for grade 1, 7.1 °C for grade 2, and 6.0 °C for grade 3, respectively, (*p* = 0.0042)]. Δ*T*-A of the circumflex artery showed similar but weaker tendency than those of LAD. Significant inverse correlations were found between Δ*T*-A and Δ*T*-R1 in each territory (*p* < 0.001).

**Conclusions:**

Antegrade delivery was less effective in situations with tight proximal lesion, especially in the LAD territory. Retrograde delivery supplemented antegrade delivery. Myocardial temperature monitoring enables us to deal with inadequate cardioplegic delivery, and is a good indicator of myocardial protection.

## Introduction

In addition to sophisticated surgical techniques, optimal myocardial protection is an essential key to achieve good results of any cardiac operations [1–4]. Cardioplegia, especially cold blood cardioplegia (CBCP), has been an important strategy to minimize intraoperative myocardial injury and continues to be in widespread use [5]. So, we have adopted combined antegrade and retrograde intermittent CBCP in all of our cardiac operations. Myocardial temperature changes are the best indicators of adequacy of cardioplegic delivery in hypothermic conditions [6, 7]. However, there are some concerns about whether CBCP can properly be delivered to myocardium uniformly in patients with coronary artery disease [7]. In this study, we aimed to elucidate whether CBCP can get over coronary artery lesions of variable severity by measuring myocardial temperature at the anterior wall of the right ventricle (RV), anterior wall of the left ventricle (LV), and postero-lateral wall of the LV, before and after antegrade and retrograde delivery of CBCP in isolated coronary artery bypass grafting (CABG).

## Methods

### Materials

From April 1991 to October 2003, we made myocardial temperature measurements in 492 of 577 patients who underwent conventional on-pump isolated CABG at our institution. The patient characteristics are summarized in Table 1. This study was approved by the local Ethics Committee, and the committee waived the need for patient consent for this data analysis in anonymous fashion.

**Table 1:** Baseline patient characteristics

*N* 492	
Age (years)	63.7 ± 9.0 (31–84)
Male/female	376/116
CAD	
No. of diseased vessel: 2.7 ± 0.5 (1–3)	
OMI±: 253/239	
LVEF (%): 54 ± 5 (17–92)	
CABG	
Elective/Urgent/Emergency: 410/22/60	
Distal Anastomosis 3.1 ± 0.8 (1–5) (A–G: 1.8 ± 0.8, SVG: 1.3 ± 1.0)	
Proximal Anastomosis: Side Clamp±: 418/74	
Pump time (min) 131 ± 43 (63–616)	
Clamp time (min) 64 ± 22 (22–172)	
Delivery of CBCP 1.8 ± 0.7 (1–5) times	1; 162 Pts.
	2; 275
	3; 46, 4; 7, 5;1
PMI: 8 (2: STEMI, 6: coronary artery spasm)	
IABP: 88 (8: PMI, 80: non-PMI (53: ACS, 27: low EF))	

### Surgical technique and myocardial protection

After institution of normothermic cardiopulmonary bypass with single aortic and venous cannulation, the ascending aorta was crossclamped and CBCP solution was administered in antegrade fashion through the aortic root, then the equal dose was given retrogradely through the coronary sinus (CS) (totally 20–25 ml/kg as an initial dose). Cooled pump blood (5–6 °C) and crystalloid solution were mixed in a ratio of 4 to 1, thereby making potassium concentration of CBCP solution higher (30 mEq/L in the first dose and 15 mEq/L in all subsequent dose). Crystalloid solution was composed of saline 112 ml, mannitol 129 ml, potassium aspartate 19 ml, and magnesium sulfate 20 ml for the first dose, and saline 347 ml, mannitol 295 ml, and potassium aspartate 22 ml for the second one and thereafter. For retrograde delivery, we used transatrial CS cannulation technique with Gundry RCSP cannula (dlp Medtronic, USA) [8]. The catheter tip was placed in a position near but not to slip off from CS ostium and CS pressure was kept around 30 mm Hg during infusion. Subsequently, 10–12.5 ml/kg of CBCP solution was administered in retrograde fashion at 20–30 min intervals. Topical cooling was not used. Before release of the aortic clamp, retrograde warm blood cardioplegia (hot shot) was administered briefly. Proximal anastomoses of saphenous vein or radial artery grafts were sutured on the aortic root using partially biting clamp during the rewarming phase of beating nonworking heart in most patients.

### Study design

We measured and recorded myocardial temperatures using Monatherm thermistor system (Mallinckrodt Ph. Mo. USA), which consisted of needle thermistor probe, thermistor module and cables connecting to a monitor display. A needle thermistor probe of 1 mm in diameter and 5 mm in length was inserted into the myocardium of the anterior RV wall, anterior LV wall and postero-lateral LV wall in turn, which correspond to a territory of the right coronary artery (RCA), the left anterior descending artery (LAD), and the circumflex artery (Cx), respectively. Myocardial temperatures were measured just before and after CBCP delivery, displayed on a monitor screen and recorded. As CBCP was delivered twice or less in a majority of patients [437 (89 %) of 492 patients], we analyzed the temperature change (Δ*T*) of the first (antegrade and retrograde) and second (retrograde) CBCP delivery.

According to preoperative coronary arteriography (CAG), we ranked severity of coronary artery stenosis as follows; grade 0 for 50 % or less, grade 1 for 75 %, grade 2 for 90 %, grade 3 for 99 % (with filling delay) or 100 % (total occlusion). In the group of coronary arteries with subtotal or total proximal occlusion, those having large, limitless collateral supply were not rated as grade 3 but the severity grade of feeding arteries. We analyzed relationships between changes in myocardial temperature and lesion severity of major coronary arteries (RCA, LAD and Cx).

Statistical analysis; Descriptive data are expressed as the mean ± standard deviation (SD) for continuous variables. Statistical analysis was performed using *t* test and ANOVA (one-way analysis of variance) for continuous variables. The correlation was calculated with the Pearson’s test. Statistical significance was defined as *p* value of <0.05.

## Results

Myocardial temperature before and after CBCP delivery and their changes subgrouped according to each territory and grade of coronary artery stenosis are summarized in Table 2. Highest temperature of the RCA territory of all stenotic grades at any point of measurement may be reflected to its exposure to room air.

**Table 2:** Myocardial temperature before and after CBCP delivery and their changes subgrouped according to each territory and grade of coronary artery stenosis

	T(°C)		Fall		Fall	(min)		Rise		Fall	N	Ratio
RCA												
Grade	Before	After A.	Δ*T*-A	After R1	Δ*T*-R1	Interval	Before R2	Δ*T*-INT	After R2	Δ*T*-R2		
0	33.2	23.3	9.9	15.8	7.6	29.8	26.2	−10.6	17.4	8.8	131	27 %
1	33.6	24	9.5	16.2	7.9	29.6	26.5	−10.7	17.4	9.2	77	16 %
2	33.8	24.9	8.9	16.4	8.4	29.6	26.7	−10.3	17.7	8.9	135	27 %
3	34	23.7	10.3	15.5	8.2	30.4	27	−11.5	17.6	9.7	149	30 %
All	33.7	23.9	9.7	15.9	8.1	29.9	26.6	−10.8	17.6	9.2	492	100 %
LAD												
Grade	Before	After A.	Δ*T*-A	After R1	Δ*T*-R1	Interval	Before R2	Δ*T*-INT	After R2	Δ*T*-R2		
0	34.1	24.5	9.7	11.9	12.6	28.8	25.5	−13.7	12.9	13.8	4	1 %
1	32.7	24.5	8.2	13.4	11.2	30.9	23.7	−10.3	15.1	8.8	70	14 %
2	32.5	25.4	7.1	14.3	11.2	29.2	23.6	−9.6	15	8.9	286	58 %
3	32	25.9	6	13.7	12.3	31	23.6	−10	14	9.4	132	27 %
All	32.4	25.4	7	14	11.5	29.9	23.7	−9.8	14.7	9.1	492	100 %
Cx												
Grade	Before	After A.	Δ*T*-A	After R1	Δ*T*-R1	Interval	Before R2	Δ*T*-INT	After R2	Δ*T*-R2		
0	31.4	19.8	11.6	14	6	31.3	22	−8.2	16.3	5.9	54	11 %
1	32	22.2	9.8	13.4	8.9	29.9	23.2	−10	15	8.1	106	22 %
2	32	23	9	13.2	9.8	29.6	22.8	−9.7	14.5	8	246	50 %
3	31.7	22.5	9.1	13	9.6	29.9	22.6	−9.7	15.1	7.3	86	17 %
All	31.9	22.4	9.5	13.3	9.2	29.9	22.8	−9.6	14.8	7.8	492	100 %

**Fig. 1:**
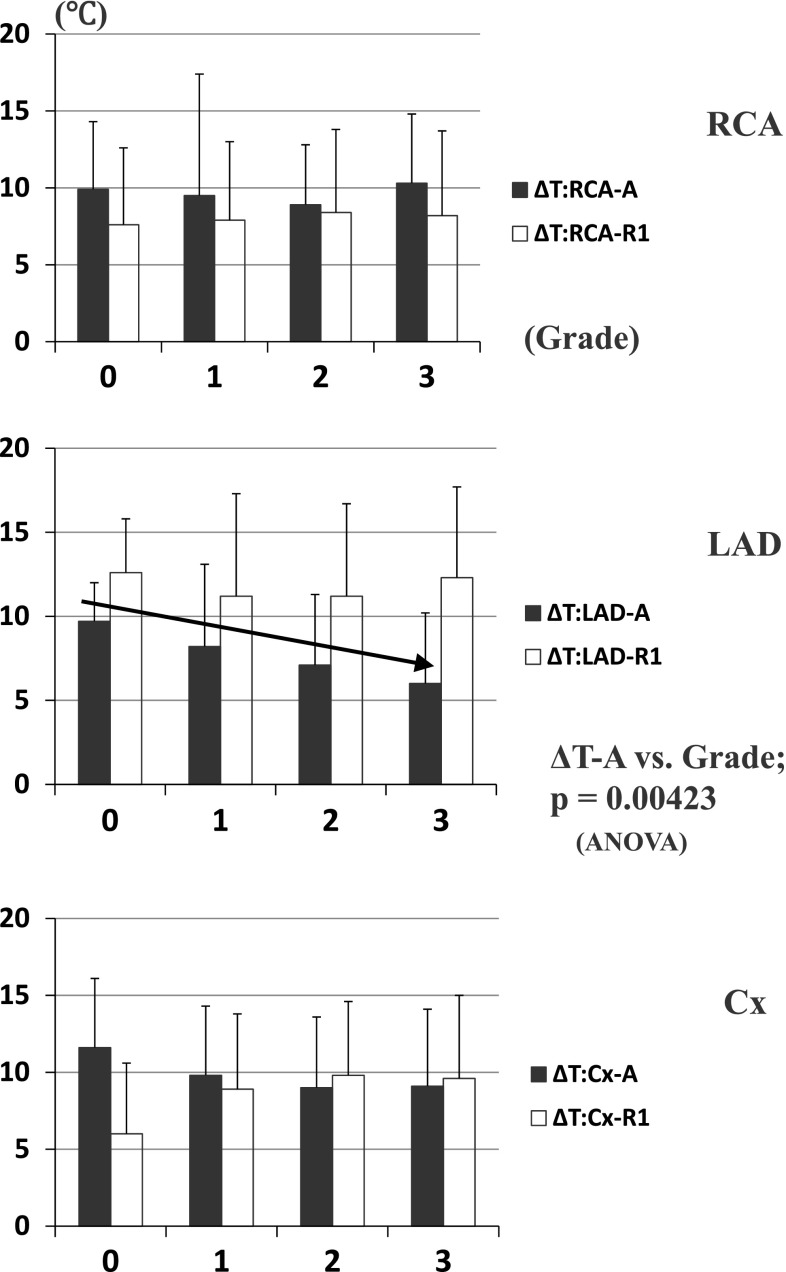
Myocardial temperature changes during initial antegrade and retrograde delivery. Bar and stick represents mean ± SD. *Upper*: RCA, *middle*: LAD, *lower*: Cx. *Black*: Antegrade, *White*: Retrograde (1st.). An *arrow* in middle graph demonstrated that Δ*T*-A of the LAD territory became less and less in proportion to its stenotic grade

Average fall in myocardial temperature during initial antegrade delivery (Δ*T*-A) and retrograde one (Δ*T*-R1) are shown in Fig. 1. In the RCA territory, mean Δ*T*-A was greater than Δ*T*-R1 irrespective of its stenotic grade, i.e., antegrade delivery was more effective than retrograde one in this area and had no relationship with stenotic grades. In the LAD territory, mean Δ*T*-A of each severity grade was 9.7 °C for grade 0, 8.2 °C for grade 1, 7.1 °C for grade 2, and 6.0 °C for grade 3, respectively (*p* = 0.0042) (Fig. 1, an arrow in middle). In contrast, Δ*T*-R1 was over 11 °C in every severity group. Thus, Δ*T*-A not only was less than Δ*T*-R1 in any stenotic grade, but also became less and less in proportion to its stenotic grade. In Cx territory, mean Δ*T*-A showed similar decreasing tendency in proportion to its stenotic grade, but it was greater than Δ*T*-R1 in stenotic grades 0 and 1, on the contrary, less in grades 2 and 3.

**Fig. 2:**
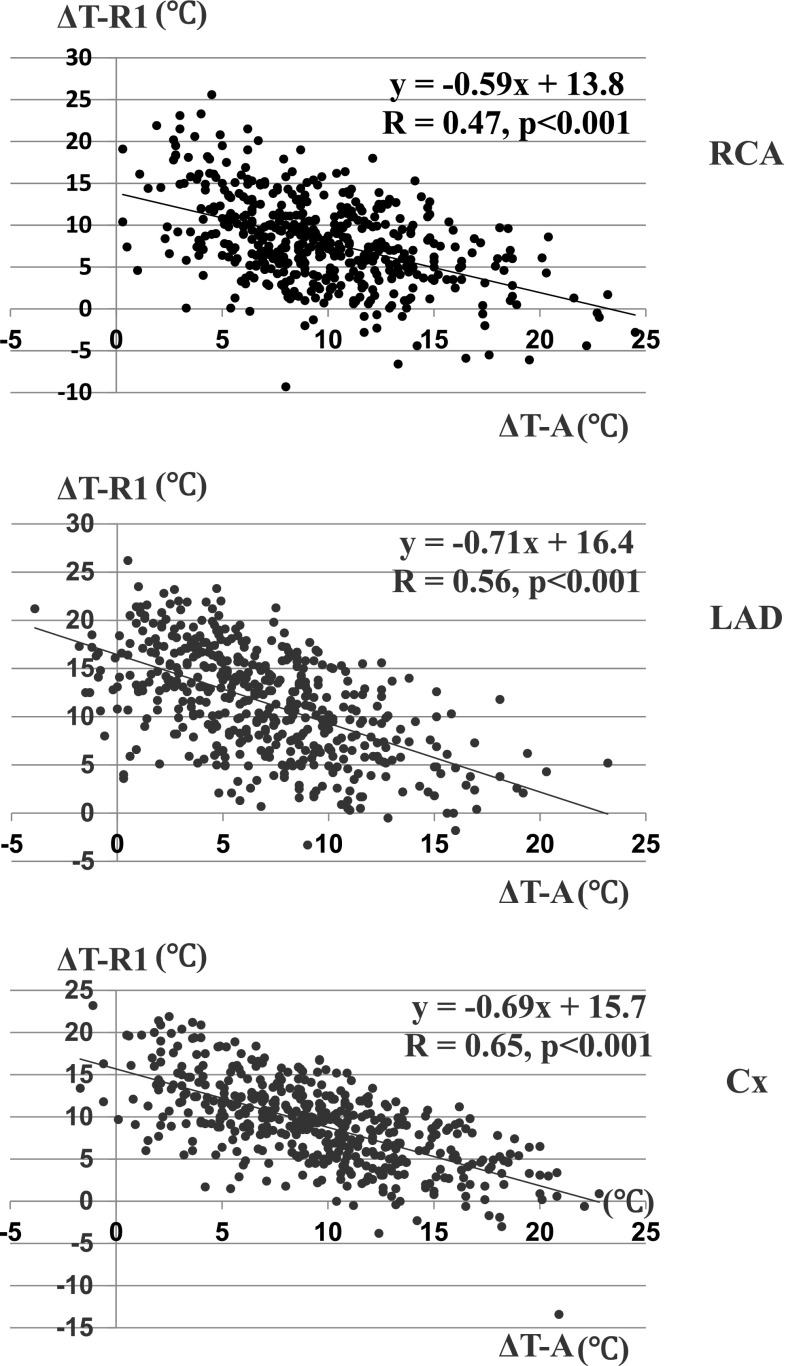
Linear regression plot of fall in myocardial temperature during initial antegrade delivery (Δ*T*-A) versus those during initial retrograde delivery (Δ*T*-R1). *Upper*: RCA, *middle*: LAD, *lower*: Cx

A representative scattergram of Δ*T*-A versus Δ*T*-R1 is shown in Fig. 2. Significant inverse correlations were observed between Δ*T*-A and Δ*T*-R1 in each territory (*p* < 0.001). This means that retrograde delivery took a role in supplementing inadequate antegrade delivery.

Rise in myocardial temperature during intervals between first and second delivery of CBCP (Δ*T*-INT) had weak correlation between Δ*T*-INT and duration of intervals in all territories. (*R* = 0.09 for RCA, 0.13 for LAD, 0.21 for Cx).

**Fig. 3:**
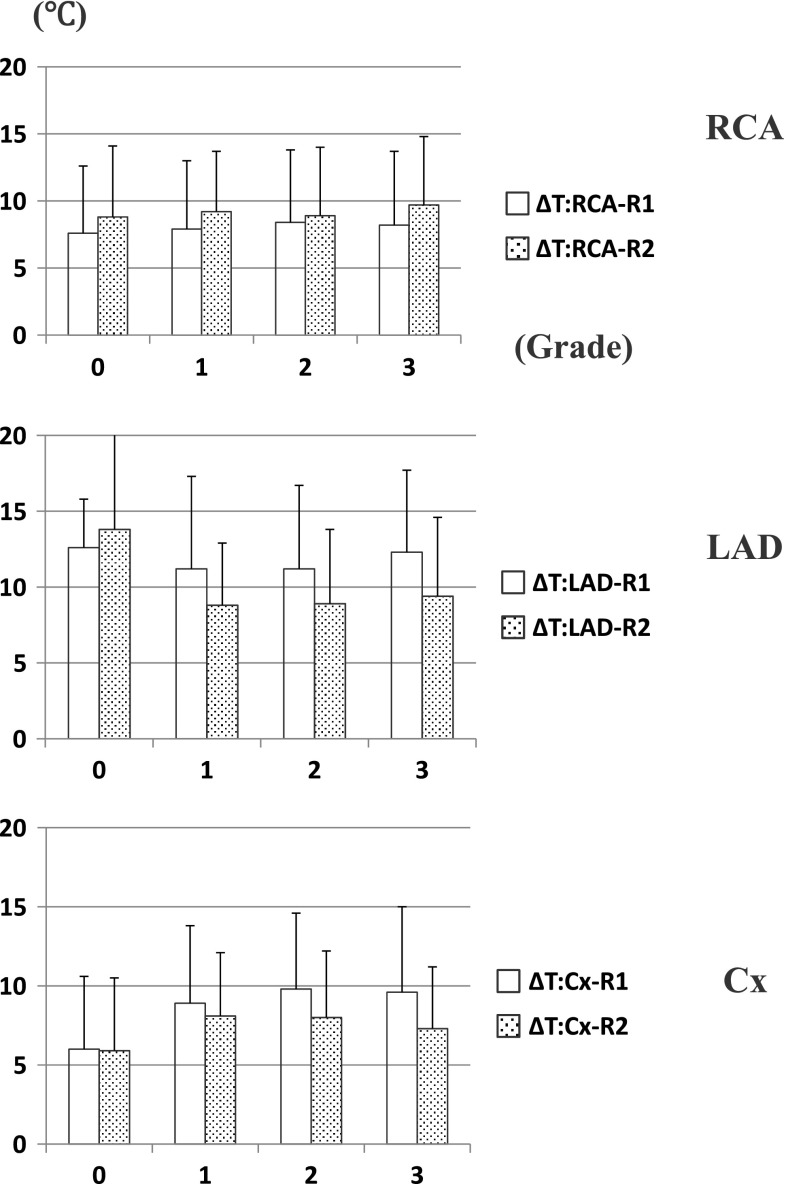
Myocardial temperature changes during second retrograde delivery. *Upper* RCA, *middle* LAD, *lower* Cx. *White* Retrograde (1st.), *Dotted* Retrograde (2nd.)

**Fig. 4:**
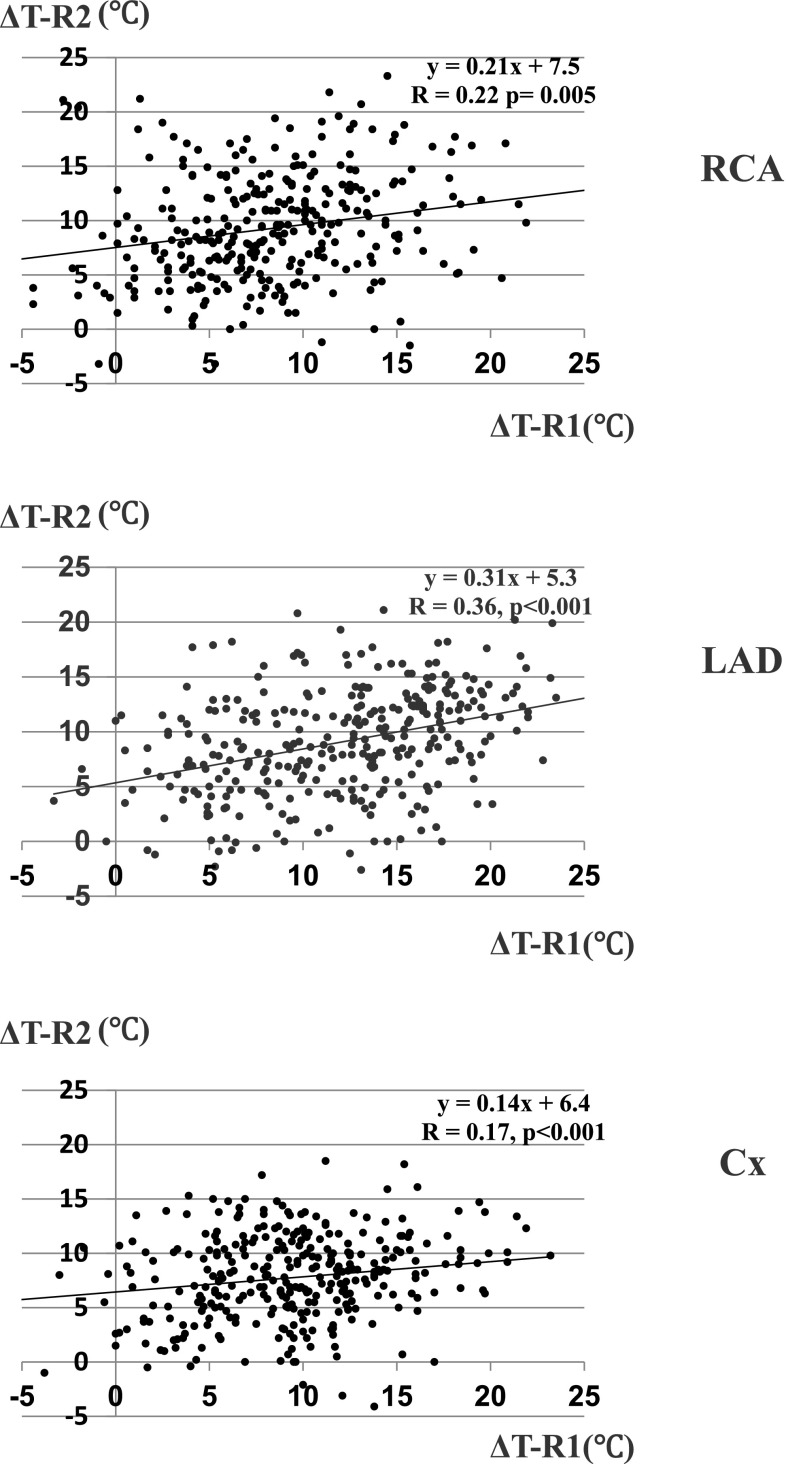
Linear regression plot of fall in myocardial temperature during initial retrograde delivery (Δ*T*-R1) versus those during second retrograde delivery (Δ*T*-R2). *Upper* RCA, *middle* LAD, *lower* Cx

Average fall in myocardial temperature during initial retrograde delivery (Δ*T*-R1) and second retrograde one (Δ*T*-R2) is shown in Fig. 3. There was no correlation between Δ*T*-R2 and stenotic grade in each territory. In comparison with fall in myocardial temperature during initial retrograde delivery (Δ*T*-R1), those during second one (Δ*T*-R2) were greater in the RCA territory, but less in the LAD and Cx territory. Gentle correlations were observed between Δ*T*-R1 and Δ*T*-R2 in each territory (Fig. 4).

Comparative analysis of myocardial temperature changes versus CBCP delivery and grade of coronary artery stenosis is summarized in Table 3.

**Table 3:** Myocardial temperature changes according to each territory and grade of coronary artery stenosis. RCA *, LAD **, Cx ***; Multiple contrasts (Turley’s test) of ANOVA on Δ*T*-A of each area

	Δ*T*-A		Δ*T*-R1		Δ*T*-R2		*p* (A-R1)	*p* (A-R1-2)
	Mean	SD	Mean	SD	Mean	SD		
RCA								
Grade 0	9.9	4.4	7.4	5.0	8.8	5.3	0.0007	NS
Grade 1	9.3	7.9	7.7	5.1	9.2	4.4	0.045	NS
Grade 2	8.9	3.9	8.4	5.4	8.7	5.1	NS	NS
Grade 3	10.2	4.5	8.1	5.5	9.6	5.1	0.0003	NS
*p* (Grade)	0.042*		NS		NS			
LAD								
Grade 0	9.7	2.3	12.6	3.2	9.2	8.0	NS	NS
Grade 1	8.1	4.9	10.9	6.1	8.7	4.1	0.0022	NS
Grade 2	7.0	4.2	11.0	5.5	8.8	4.9	0.0038	NS
Grade 3	6.0	4.2	12.2	5.4	9.4	5.2	<0.0001	NS
*p* (Grade)	0.00423**		NS		0.034			
Cx								
Grade 0	11.6	4.5	5.9	4.6	5.3	4.6	<0.0001	<0.0001
Grade 1	9.8	4.5	8.9	4.9	8.1	4.0	NS	<0.0001
Grade 2	8.9	4.6	9.6	4.8	7.8	4.2	NS	NS
Grade 3	8.9	5.0	9.4	5.4	7.3	3.9	NS	<0.0001
*p* (Grade)	0.012***		<0.0001		0.025			
*p* (Grade-all)	<0.0001		<0.0001		0.006			

* Grade 2 versus 3: *p* ≤ 0.05

** Grade 0 versus 2: *p* < 0.01, 0 versus 3: *p* < 0.001, 1 versus 3: *p* < 0.01

*** Grade 0 versus 2, 0 versus 3: *p* < 0.01

Relationships between myocardial temperature and perioperative myocardial infarction (PMI) and intraaortic balloon pump (IABP) use: Eight patients suffered from PMI with new Q wave on electrocardiogram and myocardial enzyme leakage. Two were due to ST-elevating evolving MI (STEMI) beginning preoperatively and the others were caused by native coronary artery spasm. All of them necessitated IABP support. Besides them, IABP was needed in 80 patients; two-thirds underwent emergent surgery for acute coronary syndrome not STEMI and the rest of them had failing heart due to old myocardial infarction. These events were totally unrelated to myocardial temperature changes or adequacy of CBCP delivery.

## Discussion

Cold blood cardioplegia (CBCP) has been an important strategy to minimize intraoperative myocardial injury and continues to be in widespread use [1–5], although some controversies remain about cold versus warm [9–11], blood versus crystalloid [12–14], antegrade versus retrograde [12, 15, 16], and intermittent versus continuous delivery [9]. The underlying rationale of CBCP is that during ischemic arrest, hypothermia further reduces myocardial metabolism, and oxygenated blood provides optimal buffering capacity. In addition, hypothermia allows intermittent perfusion of CBCP, thereby offering bloodless dry fields and facilitating intraoperative manipulation.

It is well recognized that myocardial temperature is the best indicator of cold cardioplegic delivery [7] and may not be uniform in the presence of coronary artery disease [6]. However, there have been no reports as to how CBCP can get to the myocardium over coronary artery lesions of variable severity. We classified stenotic severity of three major coronary arteries (RCA, LAD and Cx) into four grades according to preoperative CAG, and analyzed its relationship to myocardial temperature changes. However, subtotal or total occlusion of major coronary arteries could develop collateral channels, which might potentially relieve myocardial ischemia. So, we ranked only those with limited or lack of collateral flow (delayed or no filling on CAG) as grade 3. To our knowledge, this report is one of the first detailed reports describing such relationships.

In this study, we demonstrated that fall in myocardial temperature in the LAD territory during initial antegrade CBCP delivery (Δ*T*-A) became less and less in proportion to its stenotic grade, (*p* = 0.0042) (Table 3; Fig. 1). Similar but weaker tendency was observed in the Cx territory, but no such findings in the RCA territory. These facts proved that antegrade administration is less likely to get cardioplegic solution into myocardial segments subtended by stenotic coronary arteries, especially in the LAD territory. Significant inverse correlations between Δ*T*-A and Δ*T*-R1 in each territory showed that retrograde delivery lowered further temperatures of myocardial segments with inadequate antegrade delivery and took a role in supplementing the latter one. We think that such differences in myocardial temperature changes among three territories were related to the anatomy of the coronary arterial and venous system [17]. LAD is running along the interventricular groove and lesion sites are usually located at its proximal portion in patients who need CABG, so a spot in LV anterior wall that temperature probe is placed is always downstream to its lesion and perfusion flow is inversely proportional to the severity of lesion stenosis. On the other side, main trunks of RCA and Cx are running along the atrioventricular groove, major branches arising from there, lesion sites are variable from near the ostium to distal site, so a spot of temperature probe is not always downstream of its lesion and free wall is perfused with many branches. As the CS and great cardiac vein mainly drain blood from the LV, it is natural that retrograde cardioplegia is mainly delivered to LAD and Cx and not so much to RCA. Delivery to Cx territory might be possibly influenced by a position of the CS catheter. Accordingly, the best position of its tip is near the CS ostium, as long as it would not slip off. From these perspective, myocardial temperature at the anterior LV wall should be a surrogate monitor of global myocardial protection during combination of antegrade and retrograde administration of CBCP. Moreover that enables us to notice and deal with inadequate cardioplegic delivery quickly.

### Limitation

Myocardial temperature changes are certainly useful in monitoring CBCP delivery; however, some authors reported that they did not parallel myocardial tissue PH [7], and they may not always be a representative of myocardial metabolic state, particularly during prolonged aortic clamping. Moreover, fall in myocardial temperature (Δ*T*) through CBCP delivery could be influenced by multiple factors including lesion stenosis, CBCP volume, initial myocardial temperature, and the perfusate one. Accordingly, its adequacy should be assessed by final myocardial temperature in the end of delivery rather than Δ*T*. Myocardial temperature changes were also unable to predict PMI or IABP use as well, which were primarily determined by preoperative left ventricular function or urgent status of cardiac ischemia. Nevertheless, proper delivery of cardioplegia to entire myocardium is one of the most important factors to achieve good results of any cardiac operations. Viewed from the standpoint of risk management, we believe that it is safe and secure strategy to monitor continuously myocardial temperature at the anterior LV wall of arrested heart induced by cold cardioplegia.

## Conclusions

Antegrade CBCP delivery was less effective in situation with tight proximal stenotic lesion, especially in the LAD territory. Retrograde delivery lowered further temperatures of myocardial segments with inadequate antegrade delivery and took a role in supplementing antegrade delivery. Combination of antegrade and retrograde delivery spread cardioplegic solution to all parts of myocardium evenly. As optimal myocardial protection is one of the most important factors to achieve good results of any cardiac operations, it is advisable to monitor myocardial temperature at the anterior LV wall continuously during cardioplegic arrest.

## References

[1] Barner HB (1991). Blood cardioplegia: a review and comparison with crystalloid cardioplegia. Ann Thorac Surg.

[2] Buckberg GD (1987). Strategies and logic of cardioplegic delivery to prevent, avoid, and reverse ischemic and reperfusion damage. J Thorac Cardiovasc Surg.

[3] Buckberg GD (1995). Update on current techniques of myocardial protection. Ann Thorac Surg.

[4] Yamamoto H, Yamamoto F (2013). Myocardial protection in cardiac surgery: a historical review from the beginning to the current topics. Gen Thorac Cardiovasc Surg.

[5] Robinson LA, Schwarz GD, Goddard DB, Fleming WH, Galbraith TA (1995). Myocardial protection for acquired heart disease surgery: results of a national survey. Ann Thorac Surg.

[6] Fisk RL, Ghaswalla D, Guilbeau EJ (1982). Asymmetrical myocardial hypothermia during hypothermic cardioplegia. Ann Thorac Surg.

[7] Dearani JA, Axford TC, Patel MA, Healey NA, Lavin PT, Khuri SF (2001). Role of myocardial temperature measurement in monitoring the adequacy of myocardial protection during cardiac surgery. Ann Thorac Surg.

[8] Gundry SR, Sequiera A, Razzouk AM, McLaughlin JS, Bailey LL (1990). Facile retrograde cardioplegia: transatrial cannulation of the coronary sinus. Ann Thorac Surg.

[9] Flack JE, Cook JR, May SJ, Lemeshow S, Engelman RM, Rousou JA, Deaton DW (2000). Does cardioplegia type affect outcome and survival in patients with advanced left ventricular dysfunction? Results from the CABG patch trial. Circulation.

[10] Franke UF, Korsch S, Wittwer T, Albes JM, Wippermann J, Kaluza M, Rahmanian PB, Wahlers T (2003). Intermittent antegrade warm myocardial protection compared to intermittent cold blood cardioplegia in elective coronary surgery—do we have to change?. Eur J Cardiothorac Surg.

[11] Fan Y, Zhang AM, Xiao YB, Weng YG, Hetzer R (2010). Warm versus cold cardioplegia for heart surgery: a meta-analysis. Eur J Cardiothorac Surg.

[12] Ferguson TB, Smith PK, Lofland GK, Holman WL, Helms MA, Cox JL (1986). The effects of cardioplegic potassium concentration and myocardial temperature on electrical activity in the heart during elective cardioplegic arrest. J Thorac Cardiovasc Surg.

[13] Guru V, Omura J, Alghamdi AA, Weisel R, Fremes SE (2006). Is blood superior to crystalloid cardioplegia? A meta-analysis of randomized clinical trials. Circulation.

[14] Braathen B, Tonnessen T (2010). Cold blood cardioplegia reduces the increase in cardiac enzyme levels compared with cold crystalloid cardioplegia in patients undergoing aortic valve replacement for isolated aortic stenosis. J Thorac Cardiovasc Surg.

[15] Noyez L, van Son JA, van der Werf T, Knape JT, Gimbrere J, van Asten WN, Lacquet LK, Flameng W (1993). Retrograde versus antegrade delivery of cardioplegic solution in myocardial revascularization. A clinical trial in patients with three-vessel coronary artery disease who underwent myocardial revascularization with extensive use of the internal mammary artery. J Thorac Cardiovasc Surg.

[16] Lotto AA, Ascione R, Caputo M, Bryan AJ, Angelini GD, Suleiman M. Myocardial protection with intermittent cold blood during aortic valve operation: antegrade versus retrograde delivery. Ann Thorac Surg. 2003;76(4):1227–33; discussion 1233. 10.1016/s0003-4975(03)00840-3 14530016

[17] Ruengsakulrach P, Buxton BF (2001). Anatomic and hemodynamic considerations influencing the efficiency of retrograde cardioplegia. Ann Thorac Surg.

